# Network analysis of workplace mobbing, work–family conflict, and mental health in adults from the general population

**DOI:** 10.3389/fsoc.2025.1704409

**Published:** 2026-01-12

**Authors:** Javier Hildebrando Espinoza Escobar, Jonatan Baños-Chaparro, Tomás Caycho-Rodríguez, Fabio Cesar Saldivar Celis

**Affiliations:** 1 Vicerrectorado de Investigación, Universidad Privada Norbert Wiener, Lima, Peru; 2 Facultad de Psicología, Universidad Científica del Sur, Lima, Peru

**Keywords:** adults, mental health, work environment, work–family conflict, workplace mobbing

## Abstract

**Introduction:**

Workplace mobbing and work–family conflict represent two significant risk factors for mental health in the general adult population. In an increasingly demanding work context, these issues affect workers’ psychological well-being, impacting their productivity and quality of life.

**Objective:**

To analyze the relationship between workplace mobbing, work–family conflict, and mental health in Peruvian adults employed in both the public and private sectors.

**Materials and methods:**

A total of 345 adults participated, with a mean age of 33 years (SD = 9.4). A sociodemographic questionnaire and psychological instruments were applied. An unregularized network modeling and sex-based comparison were conducted.

**Results:**

The nodes with the highest centrality and predictability values were depressive symptoms, generalized anxiety, work–family conflict, and family–work conflict. The strongest associations were found between work–family conflict and generalized anxiety, sleep quality, and family–work conflict; between depressive symptoms and generalized anxiety and sleep quality; and between workplace mobbing, family–work conflict, and depressive symptoms. No sex differences were observed.

**Conclusion:**

The patterns of association that reflect how these factors coexist within the psychosocial environment of workers. Consequently, it is recommended that organizations implement network structure provides relevant information about the relationships between workplace mobbing, work–family conflict, and mental health. The findings highlight policies that address these associated dynamics, promote work–family balance, and offer psychological support to strengthen overall well-being in the workplace.

## 1 Introduction

Mental health in the workplace constitutes a growing challenge for society. The World Health Organization (WHO) has emphasized that work environments lacking support characterized by excessive workloads, discriminatory practices or mobbing, and limited work–life balance represent a major psychosocial risk (World Health Organization, 2024). Furthermore, it reports that approximately 15% of working-age adults suffer from some type of mental disorder, with direct impacts on global productivity, loss of workdays, and deterioration of well-being (World Health Organization, 2024). In the Peruvian context, labor informality is high. According to the National Institute of Statistics and Informatics (INEI), by the first quarter of 2025, the informality rate reached 70.7%, meaning that millions of Peruvians lack social coverage and the benefits of a formal employment relationship (Instituto Nacional de Estadística e Informática, 2025). Additionally, according to the National Superintendence of Labor Inspection (SUNAFIL), during 2023 and 2024, a total of 189 workplace mobbing complaints were reported (Superintendencia Nacional de Fiscalización Laboral, 2025). This situation increases workers’ exposure to psychosocial risks and work–family conflict. Despite regulatory advances in the country such as the ratification of ILO Convention 190 on violence and harassment in the workplace (effective since June 8, 2023) and Law No. 29783 on Occupational Safety and Health mechanisms for reporting, prevention, and reparation remain limited and little known among the working population (Sticco and Villanueva, 2022). Moreover, their effective availability is restricted primarily to the 29.3% of formal workers, leaving the vast majority without real access to these protections. While these regulations represent progress in addressing psychosocial risks, it remains a priority to understand, from an empirical and psychological perspective, how phenomena such as workplace mobbing and work–family conflict are linked to workers’ mental health. These issues constitute, today, two serious problems that affect not only workers’ quality of life but also their emotional well-being and performance (Azurín, 2025).

Workplace mobbing is one of the most insidious forms of workplace violence. It is characterized by a cumulative series of repetitive harassing behaviors in a collective, systematic, and prolonged process in which multiple individuals exert pressure and hostile communication against a person, placing them in a defenseless position and forcing their elimination from the work environment either physically (expulsion) or symbolically (discredit, isolation, loss of identity) with the goal of undermining the victim’s psychological and social integrity (Meng, 2025). It is important to distinguish mobbing from other dysfunctional interpersonal behaviors that may arise from poor soft skills, such as lack of communication, empathy, or teamwork. These behaviors, while problematic, are not classified as mobbing unless they involve intentional, persistent, and harmful actions aimed at excluding or psychologically damaging another worker (Meng, 2025). Scientific literature has documented its negative consequences on mental health, including increased risk of depressive symptoms, generalized anxiety, sleep disturbances, stress, reduced social interactions, and absenteeism (Conway et al., 2025; Erfaniyan Dabbaghnoghani et al., 2025; Kim et al., 2025; Sticco and Villanueva, 2022). In severe cases, sustained exposure to this type of violence can lead to psychotropic medication use and, in other cases, risky behaviors such as suicidal ideation and attempts, underscoring the need to address it as a public health issue (Conway et al., 2025; Kim et al., 2025).

Work–family conflict is another relevant stressor in adult life (Jie et al., 2024). This inherently bidirectional phenomenon arises when job demands interfere with family responsibilities, or conversely, when family demands hinder work performance. It can be described as the difficulty of reconciling expectations and pressures from two central domains of life: work and family (Brzykcy et al., 2024). A systematic review reported that work–family conflict is more frequent and impactful than family–work conflict, and is associated with greater psychological stress, physical and psychosomatic symptoms (fatigue, malaise), and sleep difficulties including insomnia (Brzykcy et al., 2024). Other studies reinforce these findings, linking the phenomenon to mood disorders such as depressive symptoms and generalized anxiety, which increase psychological vulnerability and reduce work performance (Espinoza, 2021; Riquelme-Segura et al., 2023).

The coexistence of workplace mobbing and work–family conflict constitutes a complex and frequent phenomenon, whose interaction may generate a highly detrimental synergistic effect on psychological well-being (Brzykcy et al., 2024). A systematic review identified that work–family conflict, family weakening, family satisfaction, marital behaviors, and family emotional exhaustion are major indicators of workplace mobbing and family functioning (Jie et al., 2024). These findings show that hostile workplace dynamics not only affect individuals personally and professionally but also erode their intimate relationships and ability to maintain a healthy balance between life domains (Kossek and Kelliher, 2022). In this sense, constant work pressure combined with the inability to adequately meet family demands creates a scenario of chronic stress that significantly increases the risk of mental health problems, reinforcing the need to understand both phenomena as interrelated processes that amplify their consequences and profoundly deteriorate psychological and relational well-being (Choudhary et al., 2023).

Among the most critical mental health indicators are suicidal ideation, generalized anxiety, depressive symptoms, and sleep quality. Suicidal ideation represents the most severe manifestation of sustained, multifactorial psychological distress and is a potential risk factor for suicide planning, attempts, and death (Conway et al., 2025; Kim et al., 2025). Generalized anxiety and depressive symptoms are highly prevalent disorders that affect workers’ functionality and quality of life (Erfaniyan Dabbaghnoghani et al., 2025; Riquelme-Segura et al., 2023). Likewise, sleep quality problems are not only common symptoms of these disorders but also exacerbate them, generating a vicious cycle of functional impairment (Erfaniyan Dabbaghnoghani et al., 2025). The relationship between these indicators and workplace stressors is complex. Previous studies have shown that workplace mobbing can trigger physiological and cognitive responses that disrupt sleep, increase anxious rumination, and foster depressive symptoms (Brzykcy et al., 2024; Conway et al., 2025; Erfaniyan Dabbaghnoghani et al., 2025). Similarly, work– family conflict, by eroding personal and social resources, can amplify threat perception and reduce coping capacities, fueling psychological distress (Sankar, 2024).

The literature also suggests possible sex differences in the experience and impact of workplace mobbing, work–family conflict, and mental health indicators. Some studies have found that women are more likely to report mobbing episodes and higher levels of work– family conflict, probably due to structural inequalities in the labor market and heavier domestic and caregiving responsibilities (Brzykcy et al., 2024; Espinoza, 2021; Riquelme-Segura et al., 2023). In terms of mental health, women tend to present higher rates of depressive symptoms, generalized anxiety, and sleep problems, while men, although reporting lower prevalence of suicidal ideation, have higher risk of suicide death (Espinoza, 2021; Kim et al., 2025; Riquelme-Segura et al., 2023). These differences underscore the importance of exploring relational patterns between variables considering sex, in order to develop more specific and effective interventions.

Theoretically, this research is framed within the Job Demands–Resources Model (JDR), which posits that job conditions can be classified into two broad categories: demands and resources (Bakker et al., 2023). Job demands are physical, psychological, social, or organizational aspects that require sustained effort and, in excess, generate physiological and psychological costs. These include workplace mobbing, workload, or work–family interference (Abdou et al., 2024; Conway et al., 2025). Job resources, in contrast, are factors that facilitate goal achievement, reduce the impact of demands, and foster personal growth, such as social support, autonomy, or recognition (Bakker et al., 2023). The imbalance between demands and resources produces a dual process: strain, linked to stress and health problems, and motivation, linked to engagement and well-being (Demerouti and Bakker, 2022; Sankar, 2024). In this context, workplace mobbing and work– family conflict represent particularly stressful demands, as they exceed individuals’ coping capacity and erode both internal and external resources (Choudhary et al., 2023; Jie et al., 2024). This translates into greater vulnerability to emotional exhaustion, anxiety, and depression. Empirical evidence shows that these phenomena do not operate in isolation but interconnect dynamically, reinforcing both distress and resource deficits (Isvoranu et al., 2022).

The network perspective adds a crucial complement to this theoretical framework. While the JD-R describes general cause–effect relationships between demands, resources, and health outcomes, the network approach allows visualization and quantification of links within a system. Instead of conceiving symptoms or factors as mere reflections of a latent construct, the network perspective understands them as interconnected nodes that influence each other (Burger et al., 2023). Thus, a depressive symptom may trigger anxiety, which in turn may intensify sleep problems, creating a feedback loop that sustains distress. Psychosocial phenomena are inherently interdependent, with multiple feedback loops and indirect connections that shape workers’ experiences and symptom development (Isvoranu et al., 2022). Accordingly, network analysis offers a methodological alternative to capture the complexity of interactions among psychological and social variables. This approach models constructs as nodes and their relationships as connections, identifying interdependence patterns and highlighting nodes with higher centrality, meaning variables that play a strategic role in the system’s overall structure (Isvoranu and Epskamp, 2023; Williams et al., 2019).

According to the scientific literature, this study is justified not only by the need to provide empirical evidence on the interaction between workplace mobbing, work–family conflict, and mental health but also by the scarcity of research in the Peruvian context analyzing this relationship from a psychological network perspective. This research aims to analyze the network structure among these factors in working adults from both public and private sectors in Metropolitan Lima, contributing to the design of preventive strategies and psychosocial interventions that promote healthier work environments.

## 2 Materials and methods

### 2.1 Participants

The research followed an associative, comparative, quantitative, and crosssectional design. The inclusion criteria were: (a) being of Peruvian nationality, (b) being between 18 and 60 years old, and (c) currently working in the public or private sector. Individuals who did not meet these criteria were excluded from the study. To calculate the sample size, a Monte Carlo (MC) simulation method for cross-sectional network models was used (Constantin et al., 2023). Considering a network based on 7 nodes, density of 0.40, sensitivity of 0.60, and statistical power of 0.99, a sample size of 300 participants was recommended.

In total, 345 Peruvian adults from the general population participated. Participation by sex was similar for women (51.3%) and men (48.7%). The mean age was 33 years (SD = 9.4), ranging from 18 to 60 years. Regarding marital status, the majority identified as single (72.5%), followed by married (24.3%), divorced (2.6%), and widowed (0.6%). In terms of educational level, most had completed university studies (40.3%), followed by completed postgraduate studies (26.4%), incomplete university studies (12.7%), among others. The most frequent employment sectors were public administration (22.3%), education and culture (20.9%), and service and commerce (13.9%), among others. The average monthly income was S/. 4,399 soles, with a range between 300 and 90,000 soles. The prevailing type of contract was indefinite-term (54.2%) and fixed-term (45.8%). Working hours were mostly full-time (81.7%), followed by part-time over 4 h (9.8%) and part-time up to 4 h (8.5%). Job tenure was 5 years or more (28.9%), 1 to 2 years (26.1%), less than 6 months (24.1%), among others. A total of 77.7% reported not having rotating shifts, while 22.3% did. As for work schedule, 76.5% worked daytime shifts, followed by mixed shifts (22.3%) and nighttime shifts (1.2%). Weekly working hours averaged 40 to 48 h (44.3%), 20 to 40 h (21.2%), 48 to 54 h (18.3%), more than 54 h (8.9%), and less than 20 h (7.3%). Likewise, regarding the number of employees at the workplace, 35.9% reported no change, while others indicated it increased slightly (32.7%), increased significantly (14.5%), decreased slightly (11.6%), or decreased significantly (5.3%).

### 2.2 Instruments

#### 2.2.1 Sociodemographic questionnaire

A brief questionnaire was administered to collect participants’ information, which included sex, age, marital status, educational level, employment sector, type of contract, work schedule, monthly income, job tenure, rotating shifts, work shift, average weekly working hours, and changes in the number of employees at the workplace.

#### 2.2.2 Luxembourg workplace mobbing scale (LWMS)

It is a brief instrument designed to assess mobbing in the workplace (Steffgen et al., 2019). The LWMS consists of 5 items, and responses range from never (1) to almost always (5), with higher scores indicating greater exposure to workplace mobbing. In this study, a Spanish translation and content validity assessment were conducted by five professional psychologists. Aiken’s V was greater than 0.70 for the criteria of relevance, clarity, and representativeness. Likewise, a confirmatory factor analysis was performed, showing adequate fit indices [CFI = 0.99, RMSEA = 0.04 (95% CI: 0.001, 0.092), SRMR = 0.02] and reliability (*ω* = 0.79).

#### 2.2.3 Work–family conflict and family–work conflict scales

It is a scale consisting of 10 items distributed across two dimensions, where the first five items assess family conflicts affecting work, while the remaining five items measure work conflicts affecting family life. Each item is rated on a seven-point Likert scale (1 = strongly disagree, 7 = strongly agree). Since it has not been adapted for the Peruvian population, a Spanish version was used for its psychometric analysis (Schetsche et al., 2022). In this study, the scale showed adequate content validity assessed by expert judgment, in which five psychologists evaluated the content based on relevance, clarity, and representativeness, obtaining and Aiken’s V greater than 0.70. In addition, two-dimensional factor structure was adequate (CFI = 0.98, RMSEA = 0.05 [95% CI: 0.037, 0.074], SRMR = 0.04) and acceptable reliability for the family–work dimension (*ω* = 0.88) and the work–family dimension (ω = 0.94).

#### 2.2.4 Frequency of suicidal ideation inventory (FSII)

It is an inventory that measures the frequency of suicidal ideation over the past year based on five items. Responses are provided on a Likert-type scale ranging from 1 (never) to 5 (almost every day). The sum of the items yields a total score ranging from 5 to 25 points. Higher scores indicate greater frequency of suicidal ideation. The Peruvian adaptation was used in this study, which showed acceptable reliability (*ω* = 0.94) (Baños-Chaparro et al., 2021a,b).

#### 2.2.5 Patient health questionnaire-2 (PHQ-2)

It is a brief questionnaire that assesses depressive symptoms over the past 2 weeks through two items. Each item is rated on a four-point scale ranging from 0 (not at all) to 3 (nearly every day). The sum of the two items provides a total score ranging from 0 to 6 points. Higher scores indicate greater depressive symptoms. The Peruvian adaptation was used, and in the present study it showed good reliability (*ω* = 0.86) (Baños-Chaparro et al., 2021a,b).

#### 2.2.6 Generalized anxiety disorder-2 (GAD-2)

It is a short-form scale that assesses generalized anxiety over the past 2 weeks through two items. The response format includes four options ranging from 0 (not at all) to 3 (nearly every day). The sum of the two items provides a total score ranging from 0 to 6 points. Higher scores indicate greater generalized anxiety. The Peruvian adaptation was used, and in the present study it showed adequate reliability (ω = 0.93) (Baños-Chaparro, 2022).

#### 2.2.7 The Jenkins sleep scale (JSS)

It consists of four items that measure the frequency and intensity of sleep quality over the past 4 weeks, including difficulty falling asleep, sleep interruptions, frequent awakenings, and feelings of fatigue upon waking despite having slept. Each item is rated on a six-point Likert scale, ranging from “it does not occur” (0) to “it occurs between 22 and 31 days” (5). The total score ranges from 0 to 20, with higher scores reflecting poorer sleep quality. The Peruvian adaptation was used, and in the present study it showed good reliability (ω = 0.85) (Palao-Loayza et al., 2024).

### 2.3 Procedure

Data collection was carried out online through a web-based survey between May and July 2025. Google Forms was used to host the survey, which was shared via the researchers’ social media. The form included information about the study’s objective, anonymity, the academic purposes of the research, data processing, and informed consent. Internet based surveys provide greater access to the study sample, systematic control of survey responses, the use of multiple channels to distribute information, and an efficient and cost effective means of administration (Gosling and Mason, 2015).

### 2.4 Data analysis

Statistical analysis was conducted using RStudio (version 4.3.2). In the initial phase, descriptive measures such as mean and standard deviation were calculated to summarize average scores. Prior to network construction, node redundancy was examined using the goldbricker function from the networktools package, identifying pairs of nodes with more than 25% topological overlap and applying a significance threshold of *p* = 0.05 (Hittner et al., 2003; Jones, 2021).

Undirected network estimation was structured into three phases. In the first phase, the estimateNetwork function from the bootnet package was used to generate an unregularized network model, employing the ggmModSelect algorithm with Spearman correlations, given its suitability for non-symmetric data (Isvoranu and Epskamp, 2023). This algorithm selects the most appropriate Gaussian graphical model from 100 random models, guided by the Extended Bayesian Information Criterion (EBIC). Unregularized approaches are appropriate when the number of participants exceeds the number of nodes and the aim is to explore edges and centrality in the network (Burger et al., 2023; Isvoranu and Epskamp, 2023; Williams et al., 2019). Network visualization was carried out using the qgraph package, applying the Fruchterman–Reingold algorithm: nodes are represented as circles and their conditional associations as lines, with blue indicating positive connections and red negative ones. Line thickness and color intensity reflect the strength of the association (Epskamp et al., 2012; Fruchterman and Reingold, 1991).

In the second phase, both local and global network properties were analyzed. At the local level, expected influence (EI) was estimated using the centrality function in qgraph, which considers the direction of connections to determine each node’s overall relevance (Epskamp et al., 2012). Predictability was also evaluated through the coefficient of determination (R^2^), computed with the predict function from the mgm package, which reflects the extent to which a node can be predicted by its direct neighbors (Haslbeck and Waldorp, 2018; Haslbeck and Waldorp, 2020). At the global level, three metrics were calculated: density (*D*), representing the average strength of connections among nodes; global transitivity (C^△^), which assesses clustering; and average path length (APL), which indicates efficiency in information spread. Additionally, the small-world index (*S*) was obtained, where values greater than 1 reflect a well-connected network with relevant clustering structures, all computed with the smallworldIndex function in qgraph (Epskamp et al., 2012; Isvoranu et al., 2022).

In the third phase, the accuracy and stability of the network were assessed using nonparametric bootstrap methods. A total of 1,000 bootstrap samples were generated with the bootnet package, calculating 95% confidence intervals for each edge (Burger et al., 2023). To analyze stability, a case-dropping bootstrap method was applied, re-estimating the network after each random deletion. The correlation stability coefficient (CS) was computed, indicating the maximum percentage of cases that can be removed without substantially affecting EI stability. A CS value greater than 0.25 is considered acceptable (Burger et al., 2023; Isvoranu et al., 2022).

Finally, in the fourth phase, a comparative analysis between the network structures of women and men was performed. Similarity between both networks was assessed using Pearson’s correlation via the cor function. A permutation test was then applied using the NCT function from the NetworkComparisonTest package, which compared two independent groups through 1,000 random permutations to test the null hypothesis (van Borkulo et al., 2022). This evaluation was based on two indicators: the maximum statistic (*M*), estimating the overall similarity of global network structures between groups, and the global strength distance (*S*_i_), which quantifies the weighted sum of absolute differences between the connections of both networks (Isvoranu et al., 2022). For both analyses, the Holm–Bonferroni correction was applied, considering differences with *p* < 0.05 as statistically significant (van Borkulo et al., 2022).

### 2.5 Ethical considerations

The study was conducted in accordance with the ethical guidelines established by the American Psychological Association (APA) and the College of Psychologists of Peru (CPsP), organizations that regulate psychology at the international and national levels (Colegio de Psicólogos del Perú, 2024; Knapp and Fingerhut, 2024). All participants signed and submitted informed consent. Participation was voluntary, the survey was conducted anonymously, and data confidentiality was guaranteed (Hilbig et al., 2022). Furthermore, the research was reviewed and approved by the ethics committee of Universidad Privada Norbert Wiener, under registration No. 0017–2025.

## 3 Results

### 3.1 Global network properties

The network showed a density of 0.129, reflecting the identification of 10 connections, all with positive associations. The nodes exhibited a clear tendency to cluster (C^△^ = 0.428), exceeding the value expected by chance (C^△^_Random_ = 0.408). Likewise, the average number of steps required for information to spread between nodes was 1.57. Finally, the *S* index was 1.205, indicating that the symptom network exhibits typical properties of a small-world system.

### 3.2 Local network properties

Regarding the descriptive measures, Table 1 shows that the highest mean and standard deviation were observed in work–family conflict (*M* = 16.09, SD = 8.75), while the lowest were found in depressive symptoms (*M* = 1.42, SD = 1.57). In the node redundancy analysis, no suggestions were indicated, as no identical node pairs were identified.

**Table 1:** Descriptive measures and local properties.

Nodes	Descriptive		Local properties	
	*M*	SD	EI	*p*
Workplace mobbing	9.24	3.32	0.34	16.7%
Work family conflict	16.09	8.75	0.95	56.5%
Family work conflict	12.34	7.09	0.80	44.1%
Suicidal ideation	6.72	3.52	0.51	35.2%
Generalized anxiety	1.54	1.66	1.06	59.6%
Depressive symptoms	1.42	1.57	1.17	59.9%
Sleep quality	8.14	4.60	0.60	33.1%

M, mean; SD, standard deviation; EI, expected influence; P, predictability. Expected influence indicates the relevance of a node within the network, considering the direction and sign of its connections, whereas predictability reflects the degree to which a node can be explained by the nodes with which it is directly connected.

**Figure 1:**
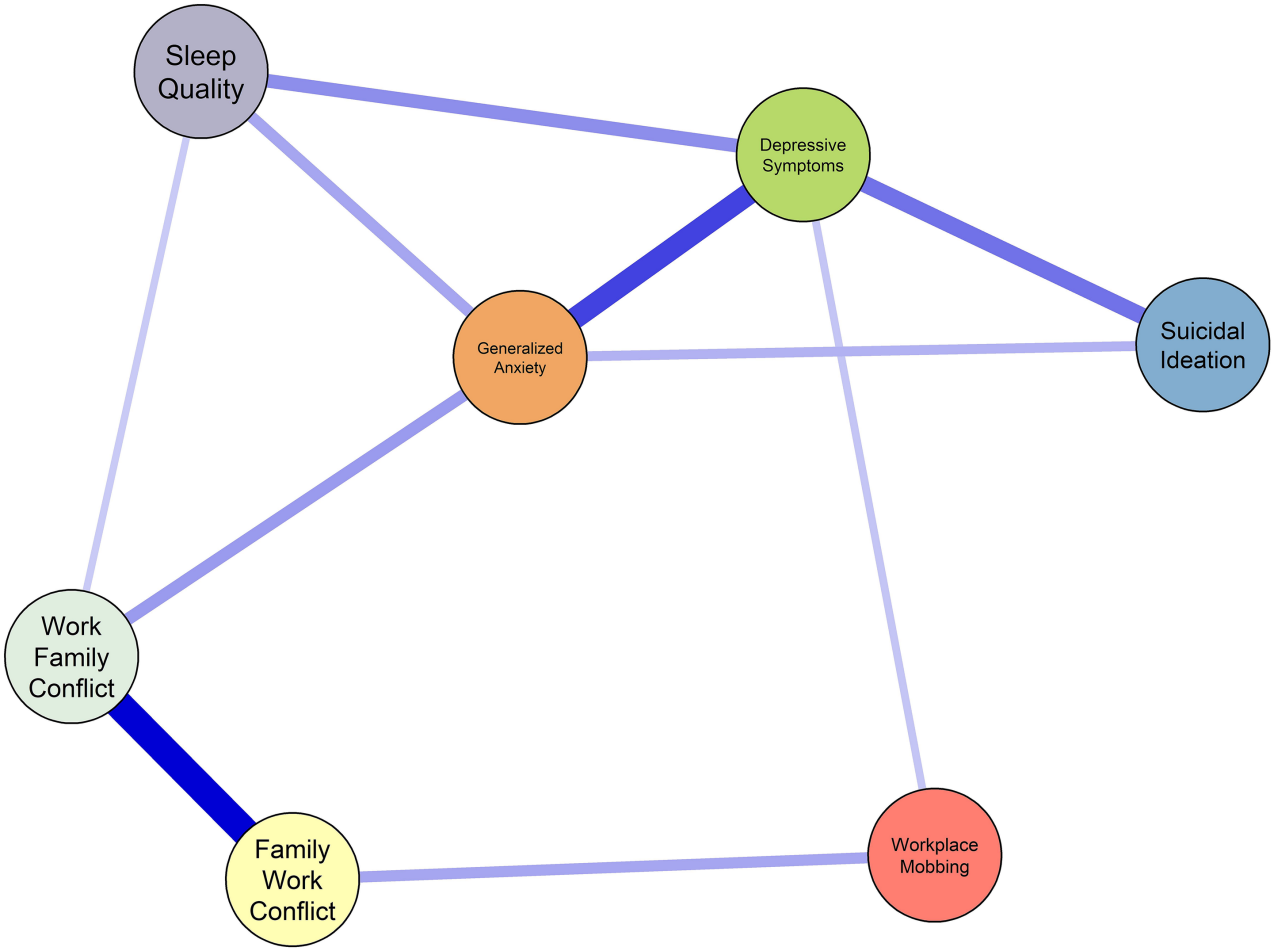
Network structure of workplace mobbing, work–family conflict, and mental health in Peruvian adults. Positive correlations are shown in blue, and negative correlations in red. The greater the intensity and thickness of the edge, the stronger the magnitude of the correlation.

With respect to local network properties, the central nodes in expected influence (EI) were depressive symptoms (EI = 1.17), generalized anxiety (EI = 1.06), work–family conflict (EI = 0.95), and family–work conflict (EI = 0.80). In terms of predictability, the nodes with the highest percentages were depressive symptoms (59.9%), generalized anxiety (59.6%), work–family conflict (56.5%), and family–work conflict (44.1%) (Table 1). Regarding the strongest relationships in the network structure, Figure 1 shows connections between work–family conflict and family–work conflict (*r* = 0.59), depressive symptoms and generalized anxiety (*r* = 0.44), depressive symptoms and suicidal ideation (*r* = 0.33), sleep quality and depressive symptoms (*r* = 0.26), generalized anxiety and work–family conflict (*r* = 0.23), family–work conflict and workplace mobbing (*r* = 0.21), workplace mobbing and depressive symptoms (*r* = 0.14), and work–family conflict and sleep quality (*r* = 0.12). The results were similar with the regularized estimation of the network structure, which can be consulted in the Supplementary materials.

### 3.3 Accuracy and stability of the network structure

**Figure 2:**
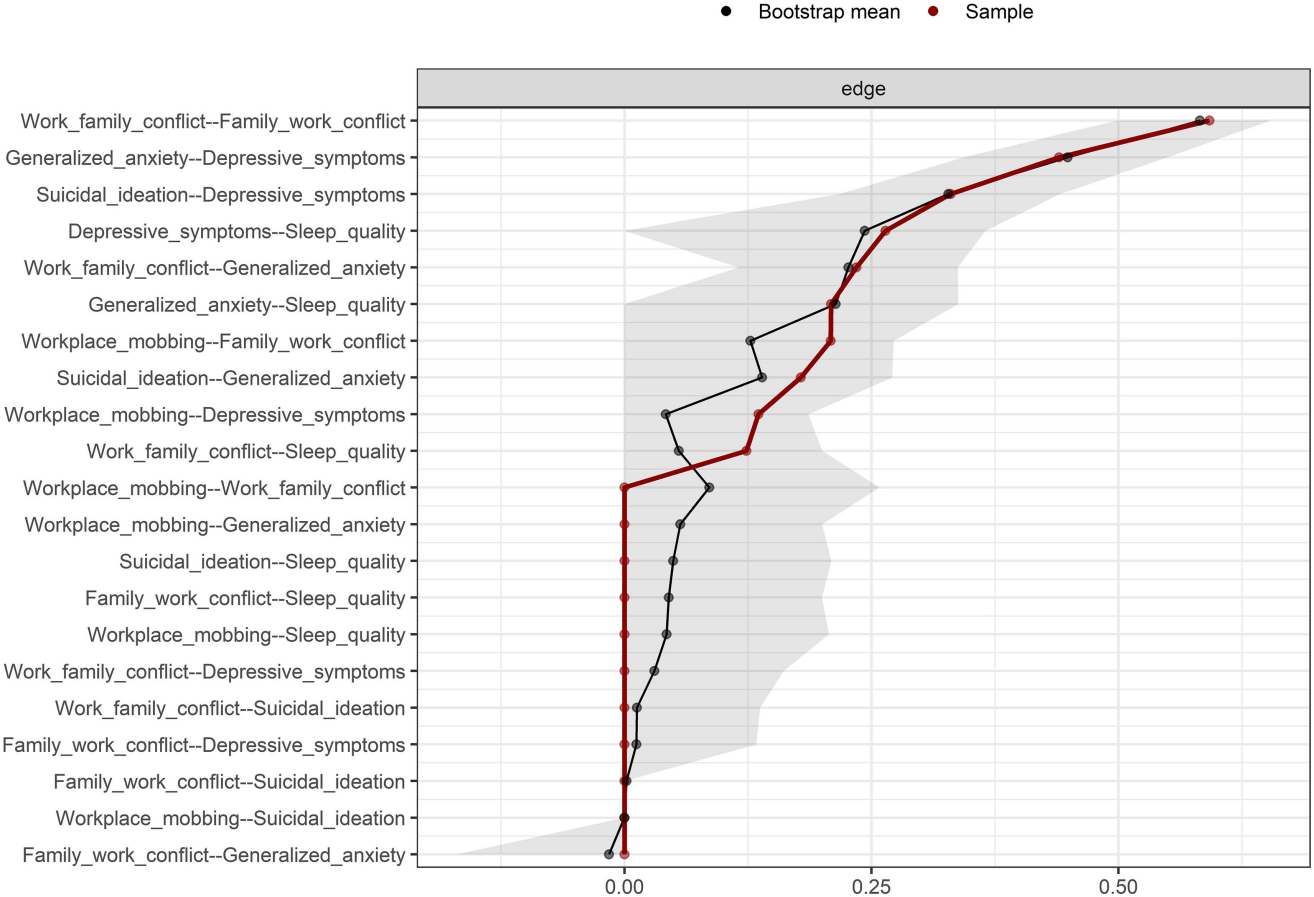
Nonparametric bootstrap confidence intervals of estimated edges for the network structure. The black line represents the sample edge. The light blue line indicates the bootstrap mean.

**Figure 3:**
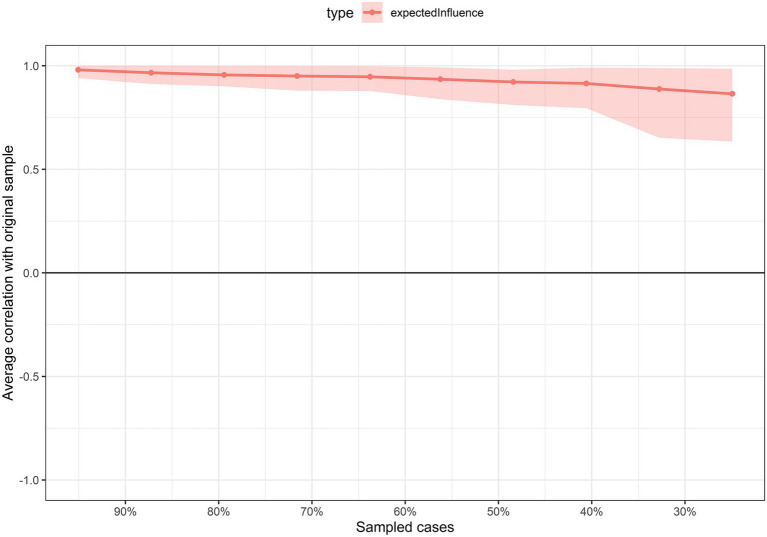
Stability of the expected influence centrality index. The light blue line indicates the average correlation of the expected influence index of the sampled network with excluded individuals and the original sample.

Figure 2 presents the accuracy of the connections. Overall, the confidence intervals (CI) around the original sample and the resampling-derived mean were narrow and consistent for most edges. Figure 3 shows the stability of EI. The analysis performed through the progressive removal of different percentages of the original sample revealed good stability [CS = 0.75 (minimum = 0.672, maximum = 1)], suggesting that the results are robust and can be interpreted reliably. The results were similar with the regularized estimation of the network structure, which can be consulted in the Supplementary materials.

### 3.4 Network comparison by sex

**Figure 4:**
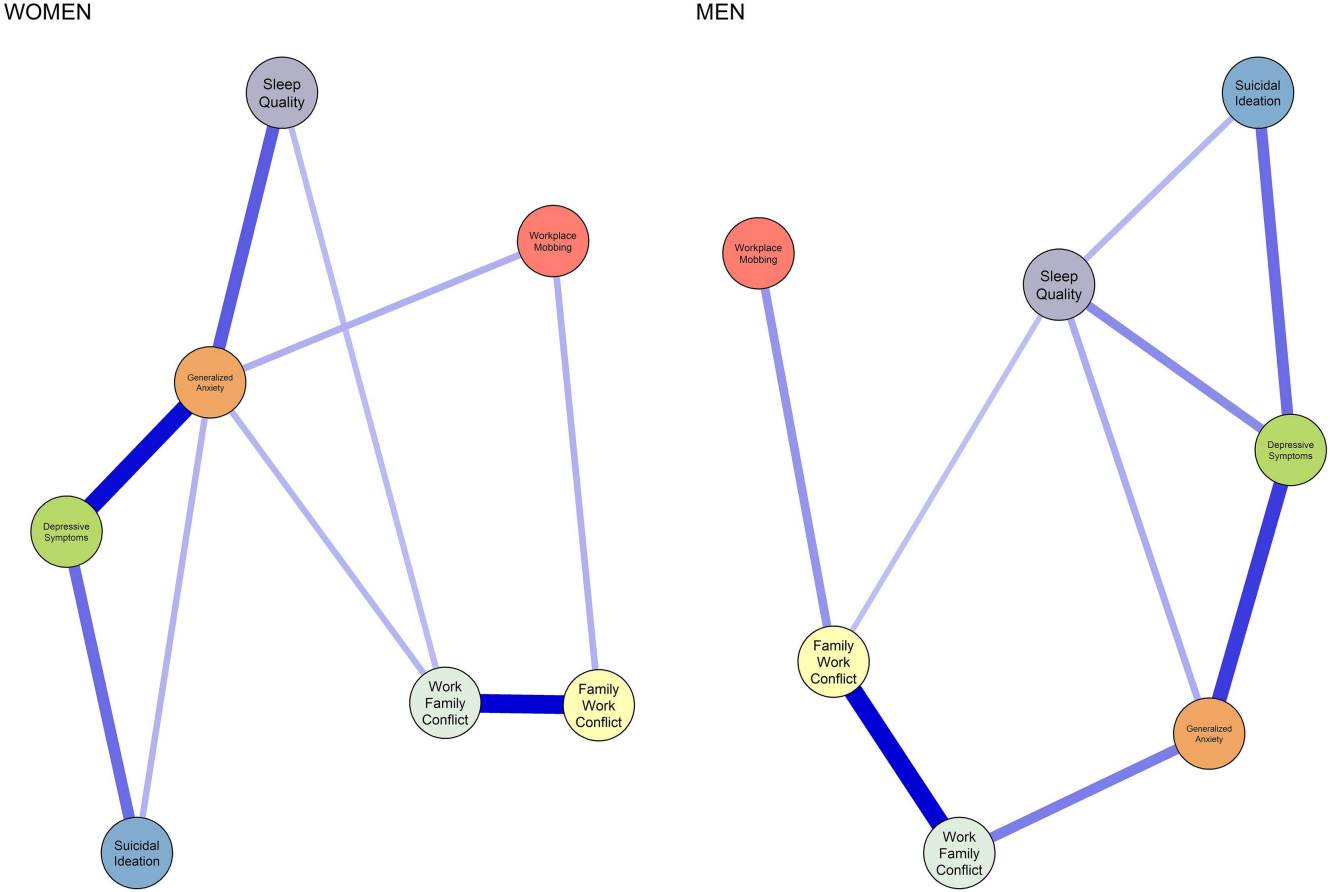
Network structure comparison by sex. Positive correlations are shown in blue, and negative correlations in red. The greater the intensity and thickness of the edge, the stronger the magnitude of the correlation.

Figure 4 shows the network configuration by sex (women = 177, men = 168). The correlation between the network structures of both groups was large (*r* = 0.79), indicating considerable similarity. No statistically significant differences were found regarding global strength invariance (*S*_i_ = 0.233, *p* = 0.389) or overall network structure (*M* = 0.137, *p* = 0.959).

## 4 Discussion

Workplace mobbing, work–family conflict, and mental health have become highly relevant and interrelated phenomena in the occupational context. Several studies indicate that workplace mobbing constitutes a psychosocial risk factor that undermines job satisfaction, productivity, and quality of life (Choudhary et al., 2023; Conway et al., 2025; Erfaniyan Dabbaghnoghani et al., 2025). Likewise, conflicts arising from the interference between work and family life create an imbalance in the psychological and physical well-being of working adults, negatively impacting mental health (Abdou et al., 2024; Brzykcy et al., 2024; Sankar, 2024). In this context, it is essential to analyze these variables in an integrated way within the general population, particularly in Latin American settings such as Peru, where working conditions and family demands often coexist under high levels of informality and precariousness. The objective of this study was therefore to understand how these psychological variables interrelate through a network analysis, which allows for identifying the most influential nodes and strongest connections, as well as differences by sex.

The results showed that depressive symptoms, generalized anxiety, and work–family and family–work conflicts constituted the nodes with the greatest influence and predictability. This finding is noteworthy as it underscores the structural role of these symptoms and inter-role tensions within the psychosocial network analyzed. In the literature, it has been highlighted that the centrality of emotional symptoms functions as a bridge linking contextual stressors such as workplace mobbing or work–family conflicts with mental health outcomes, thereby reinforcing a vicious cycle of psychological vulnerability (Conway et al., 2025; Brzykcy et al., 2024; Isvoranu et al., 2022). In other words, depressive and anxious symptoms not only reflect the impact of the environment but are also closely associated with greater distress. A possible explanation for this dynamic is the cumulative nature of exposure to chronic stressors. Workplace mobbing and conflicts between work and family domains do not occur in isolation but interact continuously and are associated with reduced emotional and cognitive resources, as well as greater sensitivity to threat (Choudhary et al., 2023; Jie et al., 2024). This erosion contributes to between work and family domains do not occur in isolation but interact continuously and heightened sensitivity to threat and daily tensions, which in turn reinforces negative perceptions of both the workplace and the home environment.

The strongest correlations were observed between depressive symptoms and generalized anxiety, suicidal ideation, and sleep quality. These findings are particularly relevant, as they reflect patterns consistent with the clinical literature that acknowledges the high comorbidity between depression and anxiety, as well as the role of depression as one of the most robust predictors of suicidal ideation (Erfaniyan Dabbaghnoghani et al., 2025; Kim et al., 2025; World Health Organization, 2024). In the context of the Peruvian adult population employed in public and private sectors, this relationship acquires additional importance, as it suggests that the emotional burden derived from adverse work environments can have severe consequences for mental health, extending beyond everyday distress (Azurín, 2025; Instituto Nacional de Estadística e Informática, 2025). Likewise, correlations between anxiety and work–family conflict, workplace mobbing and depressive symptoms, as well as between family–work conflict and workplace mobbing, and work–family conflict and sleep quality, suggest that interpersonal and occupational tensions are associated with higher levels of stress and emotional dysregulation. In this regard, the results indicate that adults working in emotionally demanding contexts, marked by mobbing, overload, and difficulties in balancing work and family roles, are particularly exposed to mental health deterioration (Choudhary et al., 2023; Conway et al., 2025; Jie et al., 2024). From the JD-R perspective, these correlations can be explained through the health impairment process: workplace mobbing and inter-role conflicts may represent excessive job demands associated with lower physical and emotional resources and with higher levels of anxiety and depressive symptoms and sleep disturbances (Abdou et al., 2024; Demerouti and Bakker, 2022). The lack of adequate resources limits workers’ ability to compensate for these demands, facilitating progression toward emotional exhaustion and greater risk of suicidal ideation (Bakker et al., 2023; Kim et al., 2025).

The finding of no significant sex differences in the network structure contrasts with part of the literature reporting greater vulnerability among women to the effects of work–family conflict. This contrast is relevant, as it suggests that the patterns of influence of occupational and family stressors may be shifting in certain segments of the Peruvian population. One possible explanation is that sociocultural transformations in gender role distribution have begun to balance, at least partially, the perceived burdens between working men and women (Brzykcy et al., 2024; Kossek and Kelliher, 2022). In urban contexts, with greater access to higher education and more equitable employment opportunities, progressive coresponsibility in domestic and caregiving tasks is observed, which may dilute the differences traditionally reported in the international literature (Espinoza, 2021; Perreault and Power, 2021; Riquelme-Segura et al., 2023). Another plausible explanation relates to the characteristics of the analyzed sample. The predominance of adults with university and postgraduate education likely reflects a group embedded in more homogeneous work and family environments in terms of role expectations and responsibility distribution. These workers tend to operate in organizational settings with greater formality, access to work–family balance policies, and negotiation opportunities in managing job demands, which reduces the gender gap in perceptions of work–family conflict (Kossek and Kelliher, 2022; Perreault and Power, 2021). However, this finding should not be generalized uncritically: in less privileged sectors or among individuals with lower educational attainment, gender differences may persist more strongly due to traditional role allocation patterns and limited opportunities to balance work and domestic demands.

The implications of this research are presented from both theoretical and practical perspectives. Theoretically, this study reinforces the utility of network analysis as a tool to understand psychopathology and occupational psychosocial risks from a dynamic perspective. By identifying central emotional symptoms, the results show that these nodes are not only clinical manifestations but also convergence points linking job and family demands with psychological well-being. In this sense, network analysis complements the JD–R framework by providing a structural and relational perspective that visually and quantitatively maps how job demands (such as workplace mobbing and work–family conflict) interact with psychological outcomes and with each other. While the JD–R model explains the processes through which demands and resources influence well-being, network analysis reveals the interconnections among these variables, allowing for the identification of central nodes that may act as mechanisms within the JD–R health impairment process. This integrative approach thus bridges theoretical and empirical levels, enhancing the explanatory power of the JD–R framework. Furthermore, from the JDR model, the findings support the idea that emotional symptoms function as key indicators of the health impairment process, in which excessive demands such as workplace mobbing and work–family conflicts are linked to lower personal resources and higher levels of persistent distress (Bakker et al., 2023; Demerouti and Bakker, 2022). Practically, the findings underscore the urgency of interventions aimed at improving sleep quality, preventing and treating depressive and anxious symptoms, and designing organizational strategies to reduce workplace mobbing and work–family and family–work conflicts. These recommendations derive directly from the network results, in which depressive symptoms, generalized anxiety, and work–family conflicts emerged as the most central and predictable nodes. Therefore, interventions should prioritize these variables as key targets for reducing the overall connectivity and intensity of the psychosocial network, potentially weakening the links that sustain mental health deterioration. In particular, improving sleep quality may buffer the reciprocal associations between emotional distress and work demands identified in the model, while organizational actions against mobbing could decrease the propagation of stress throughout the network. In the Peruvian context, where labor informality exceeds 70% and institutional support systems are limited, these implications become a priority (Azurín, 2025; Instituto Nacional de Estadística e Informática, 2025). In the public sector, it is crucial for institutions to implement accessible and sustainable workplace well-being programs, integrating psychological support services and work–family balance strategies. In the private sector, particularly in medium and large companies, the development of awareness campaigns on workplace mobbing, clear prevention and intervention protocols, and flexible work policies to mitigate inter-role tensions are recommended. At the level of public policy, it is necessary to recognize the impact of workplace mobbing and work–family conflicts as psychosocial risks that directly affect productivity and the mental health of working adults. This entails advancing regulations that promote organizational climate monitoring, encourage work–family reconciliation, and strengthen mental health coverage in public services.

The main strength of this study lies in the application of network analysis, an innovative methodology that captures the interdependence among symptoms and psychosocial factors through a system of nodes and edges, compared to traditional models, providing a dynamic view of work-related mental health processes. However, several limitations must be acknowledged. First, the non-probabilistic sampling and the relatively modest sample size restrict the generalizability of the findings to the entire Peruvian workforce. Future studies should employ probabilistic or stratified sampling strategies to include greater sectoral and regional diversity. Second, the cross- sectional design prevents establishing causal relationships among variables; therefore, longitudinal research is recommended to examine the directionality of the identified links and the cumulative effect of workplace mobbing, family demands, and mental health. Third, the overrepresentation of participants with university and postgraduate education may have biased the findings toward a population with greater cognitive and occupational resources. Future research should broaden the scope to workers with lower educational attainment and in more vulnerable conditions, where the impact of mobbing and inter-role conflicts may differ. Finally, the inclusion of a limited set of variables excludes protective factors such as resilience, social support, or coping strategies, which could act as key resources within the JD-R model. Incorporating these variables in future studies would allow for a better understanding of buffering mechanisms against work and family demands. Additionally, it should be noted that only Peruvian citizens were evaluated in this study, and no data were collected from non-citizens or migrant workers. Future research should consider the inclusion of foreign residents, as migration background and cultural legacy could influence the perception and experience of workplace mobbing, work–family conflict, and mental health.

## 5 Conclusion

In conclusion, this study highlights the centrality of depressive symptoms, generalized anxiety, and work–family and family–work conflicts in the network of workplace mobbing and mental health among Peruvian adults. The strongest correlations between depressive symptoms, generalized anxiety, suicidal ideation, and sleep quality, along with their association with workplace mobbing and work–family conflicts, suggest that emotional distress is associated with a network of mutually reinforcing symptoms and occupational tensions. The absence of sex differences suggests that cultural and contextual factors may be shaping these dynamics. Overall, the findings reinforce the importance of mental health in the general adult population, with the aim of preventing and reducing psychosocial risks in public and private occupational settings in Peru.

From an applied perspective, the results allow for the identification of precise interventions. For example, efforts should focus on strengthening the early detection and management of depression and anxiety; promoting organizational policies that support work–family balance; and preventing and sanctioning workplace mobbing as a strategy to reduce the spread of stress within occupational systems. Moreover, the relationship between sleep quality and emotional distress highlights the importance of rest and recovery programs. Overall, these results provide empirical evidence to guide targeted interventions on the central nodes of the network structure, helping to reduce emotional distress and strengthen the mental well-being of the working population in the Peruvian context.

## Data Availability

The raw data supporting the conclusions of this article will be made available by the authors, without undue reservation.
