# Supplementary material for: Network analysis of workplace mobbing, work–family conflict, and mental health in adults from the general population
Source: Front Sociol. 2026 Jan 12;10:1704409. doi: 10.3389/fsoc.2025.1704409 (PMC12833962; doi:10.3389/fsoc.2025.1704409)
Supplement: Supplementary file 1 [file Data_Sheet_1.docx]

**SUPPLEMENTARY MATERIAL**

**NETWORK STRUCTURE WITH REGULARIZED METHOD (EBICglasso)**

# Network structure

In Figure 5S, the regularized estimation of the network structure can be observed. In particular, the strongest relationships emerged between work–family conflict and family– work conflict (r = 0.56), depressive symptoms and generalized anxiety (r = 0.44), depressive symptoms and suicidal ideation (r = 0.30), sleep quality and depressive symptoms (r = 0.23), generalized anxiety and work–family conflict (r = 0.20), generalized anxiety and sleep quality (r = 0.20), suicidal ideation and generalized anxiety (r = 0.15), and family–work conflict and workplace mobbing (r = 0.13).


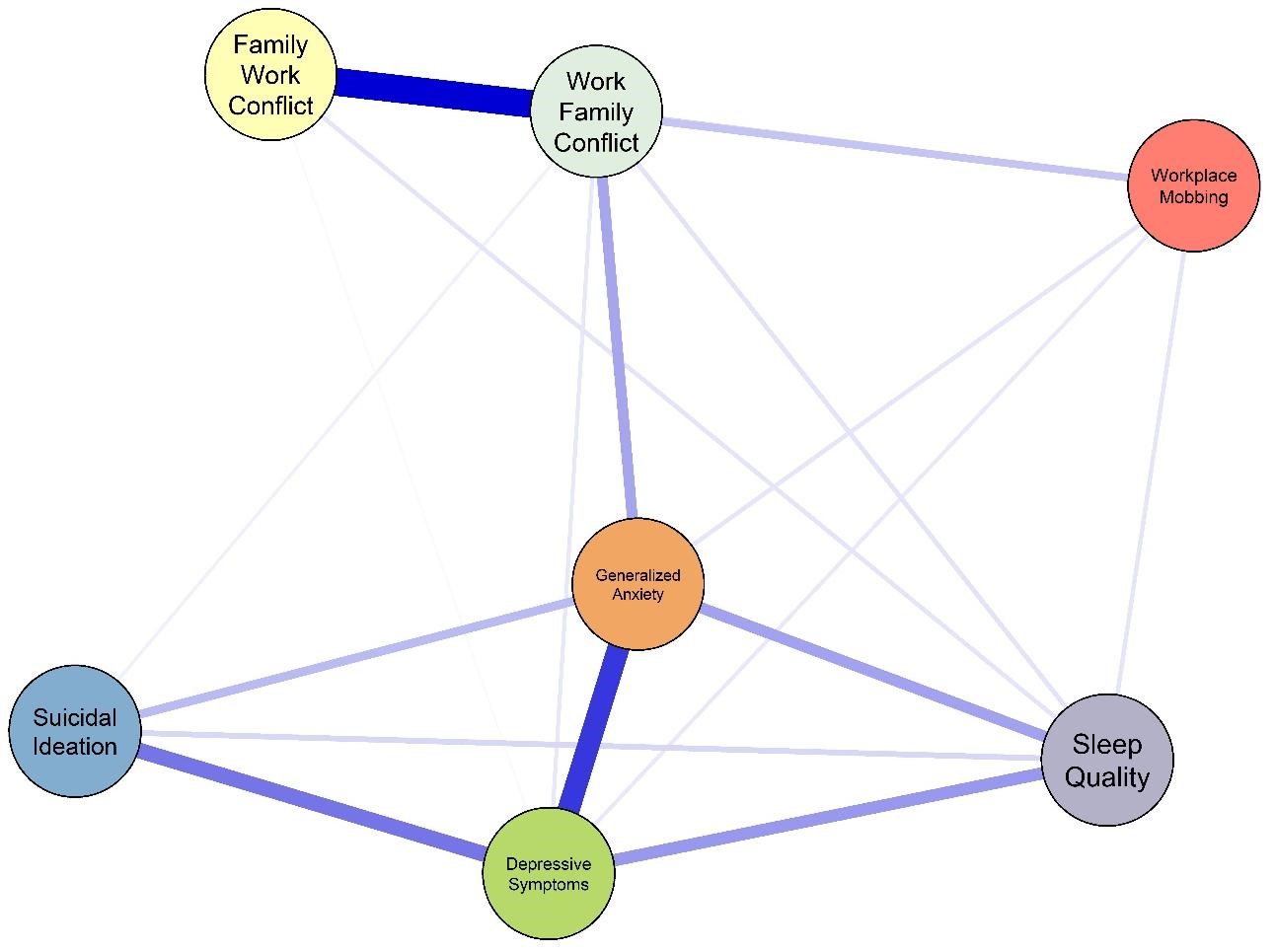


# Figure 5S

Network structure of workplace mobbing, work–family conflict, and mental health in Peruvian adults. Positive correlations are shown in blue, and negative correlations in red. The greater the intensity and thickness of the edge, the stronger the magnitude of the correlation.

# Precisión y estabilidad de la red

Figure 6S illustrates the precision of the connections. In general, the confidence intervals (CI) surrounding both the original sample and the resampling-based mean were narrow and largely consistent across most edges. Figure 7S depicts the stability of the EI. The analysis, which involved the gradual exclusion of varying proportions of the original sample, indicated high stability (CS = 0.751 [minimum = 0.672, maximum = 1]), suggesting that the findings are robust and can be interpreted with confidence.


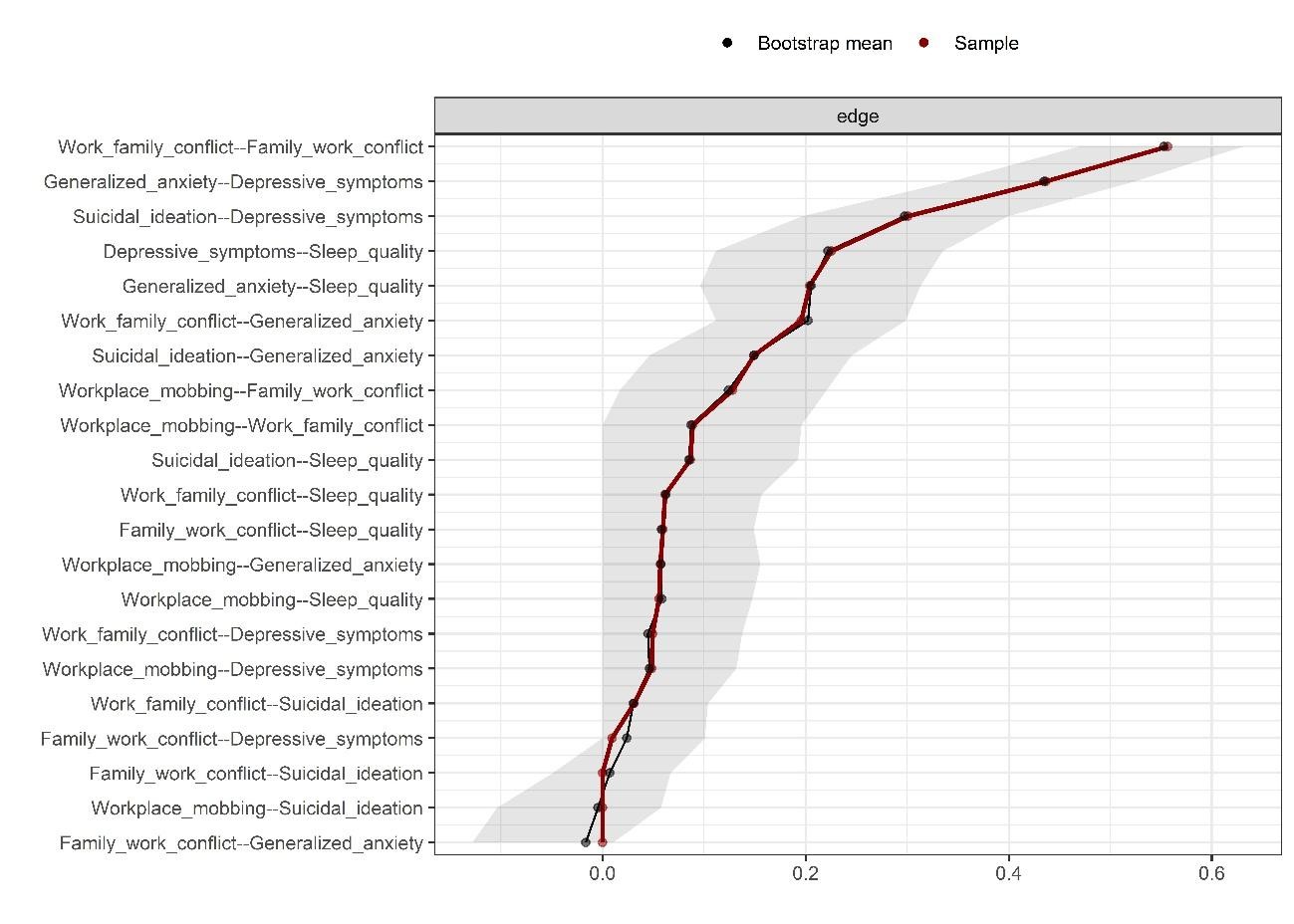


# Figure 6S

Nonparametric Bootstrap Confidence Intervals of Estimated Edges for the Network Structure. The black line represents the sample edge. The light blue line indicates the Bootstrap mean.


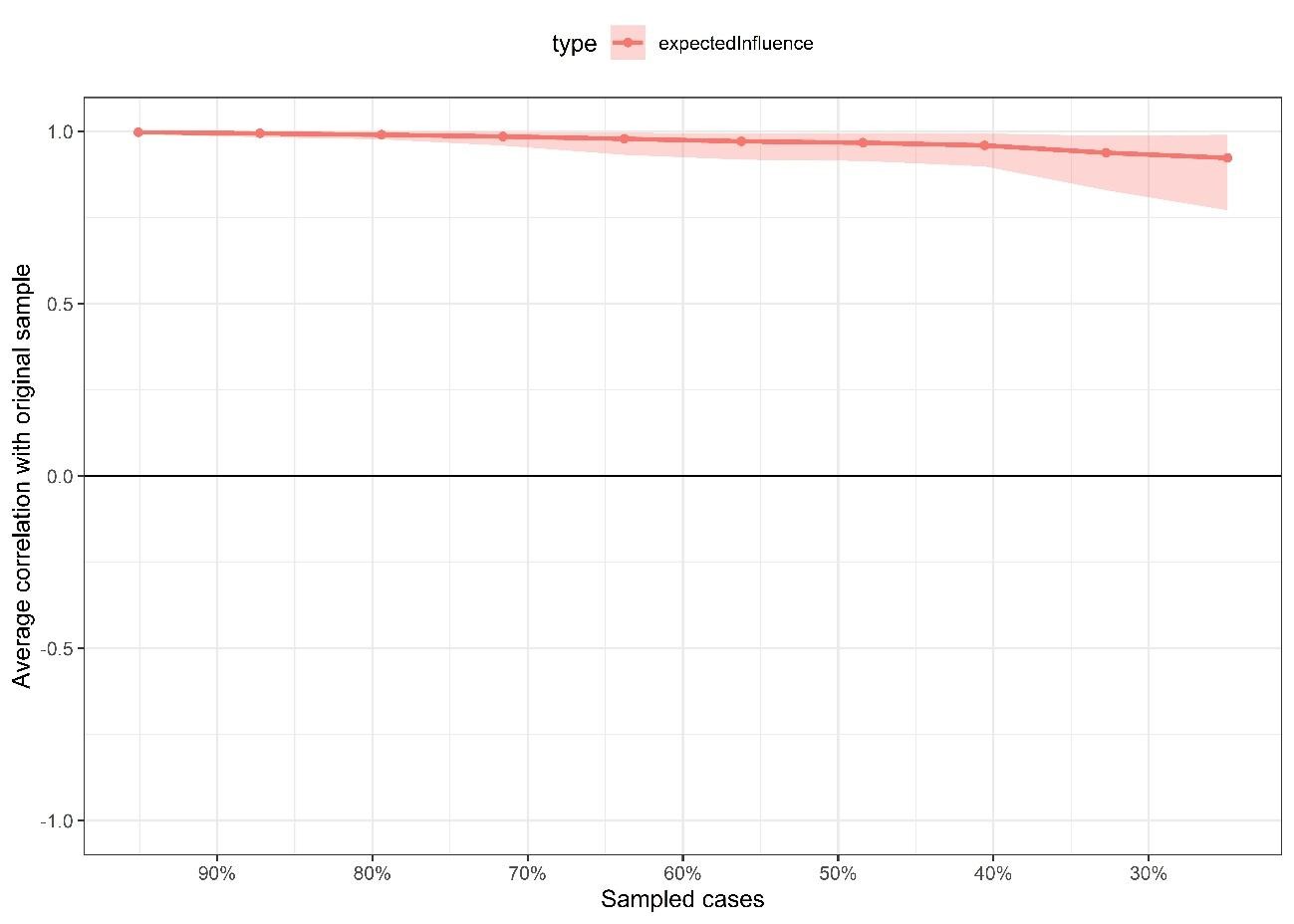


# Figure 7S

Stability of the Expected Influence Centrality Index. The light blue line indicates the average correlation of the expected influence index of the sampled network with excluded individuals and the original sample.
